# A porcine model of hemodialyzer reactions: roles of complement activation and rinsing back of extracorporeal blood

**DOI:** 10.1080/0886022X.2021.2007127

**Published:** 2021-12-09

**Authors:** Ákos Pethő, Dorothea Piecha, Tamás Mészáros, Rudolf Urbanics, Christoph Moore, Bernard Canaud, László Rosivall, Tom Eirik Mollnes, Sonja Steppan, Gábor Szénási, János Szebeni, László Dézsi

**Affiliations:** a Department of Internal Medicine and Oncology, Semmelweis University, Budapest, Hungary; b Fresenius Medical Care Deutschland GmbH, Bad Homburg, Germany; c SeroScience Ltd, Budapest, Hungary; d School of Medicine, Montpellier University, Montpellier, France; e International Nephrology Research and Training Center, Institute of Translational Medicine, Semmelweis University, Budapest, Hungary; f Department of Immunology, Oslo University Hospital, Rikshospitalet, Oslo, Norway; g Research Laboratory, Nordland Hospital Bodø and Faculty of Health Sciences and TREC, University of Tromsø, Tromsø, Norway; h Centre of Molecular Inflammation Research, Norwegian University of Science and Technology, Trondheim, Norway; i Nanomedicine Research and Education Center, Institute of Translational Medicine, Semmelweis University, Budapest, Hungary

**Keywords:** Hemodiafiltration, hypersensitivity reactions, CARPA, polysulfone membrane, pulmonary hypertension, anaphylatoxins

## Abstract

Hemodialysis reactions (HDRs) resemble complement-activation-related pseudoallergy (CARPA) to certain i.v. drugs, for which pigs provide a sensitive model. On this basis, to better understand the mechanism of human HDRs, we subjected pigs to hemodialysis using polysulfone (FX CorDiax 40, Fresenius) or cellulose triacetate (SureFlux-15UX, Nipro) dialyzers, or Dialysis exchange-set without membranes, as control. Experimental endpoints included typical biomarkers of porcine CARPA; pulmonary arterial pressure (PAP), blood cell counts, plasma sC5b-9 and thromboxane-B2 levels. Hemodialysis (60 min) was followed by reinfusion of extracorporeal blood into the circulation, and finally, an intravenous bolus injection of the complement activator zymosan. The data indicated low-extent steady rise of sC5b-9 along with transient leukopenia, secondary leukocytosis and thrombocytopenia in the two dialyzer groups, consistent with moderate complement activation. Surprisingly, small changes in baseline PAP and plasma thromboxane-B2 levels during hemodialysis switched into 30%–70% sharp rises in all three groups resulting in synchronous spikes within minutes after blood reinfusion. These observations suggest limited complement activation by dialyzer membranes, on which a membrane-independent second immune stimulus was superimposed, and caused pathophysiological changes also characteristic of HDRs. Thus, the porcine CARPA model raises the hypothesis that a second “hit” on anaphylatoxin-sensitized immune cells may be a key contributor to HDRs.

## Introduction

Renal replacement therapy, represented mainly by hemodialysis (HD), provides life-sustaining treatment for the expanding Stage 5 chronic kidney disease population worldwide. Despite technical advances and progresses in biocompatibility material, HD procedures remain associated with a relatively low risk of acute allergy-like, so-called HD-reactions (HDRs), but that occasionally may lead to life-threatening conditions within minutes after hooking the patients on an extracorporeal circuit. Over the last few years an increasing number of such hypersensitivity reactions (HSRs) have been reported with hemodialyzers, creating a new threat in the nephrology community [1–8].

Clinical manifestations of these acute adverse events include itching, burning sensation at the access site, urticaria, flushing, cough, sneezing, wheezing, abdominal cramps, diarrhea, headache, back and chest pain, nausea, vomiting, fever and chills. The most common symptoms are chest and back pain, dyspnea, nausea, vomiting and hypotension, typically occurring within 15–30 min after launch of dialysis, and depending on severity, may or may not require discontinuation of hemodialysis treatment. In the former case, requiring instant suspension of dialysis, the patient is at risk for developing anaphylactic shock with dyspnea, hypotension and sudden cardiac death.

Hypersensitivity reactions, in general, may arise *via* IgE-dependent and IgE-independent pathways, referred to as “immune”, or “true” allergy, and non-immune, or “pseudoallergy” [9]. Regarding the mechanism of HDRs, IgE-mediated reactions can arise because of leaching off some non-natural components of the extracorporeal circuit, for which the patient had been pre-sensitized. The frequency of such appliance-specific reactions is extremely rare, 1/400,000, while nonspecific HDRs have a much greater incidence (3–5/100) [10]. The latter reactions are also less severe and are thought to be mediated by activations of the complement and/or the kallikrein/kinin systems [11]. Importantly, the clinical picture of these pseudoallergic reactions is identical to that of HSRs observed with other therapies using extracorporeal circuits, namely cardiopulmonary bypass [12–14] and apheresis (hemapheresis) [15–17]. Moreover, of particular interest for the present study, these symptoms are also very similar, or identical to those of infusion reactions triggered by a variety of i.v. administered nanomedicines, contrast agents and biologicals. Because of the causal role of complement activation, these reactions have been referred to as complement activation-related pseudoallergy (CARPA) [18,19].

Pigs provide a sensitive model for nanomedicine-induced CARPA, the most quantitative and reproducible endpoints of which are hemodynamic changes, most importantly the rise of pulmonary arterial pressure (PAP). In addition, typical complement-mediated HSRs are associated with leukopenia followed by secondary leukocytosis and rises of plasma thromboxane A2 (TXA2), which is measured *via* its stable metabolite, TXB2. Leukopenia followed by compensatory leukocytosis is also typical of HDRs, and the main symptoms of severe human HSRs can be explained, in part, by pulmonary hypertension and/or other hemodynamic changes, also observed in porcine CARPA. Yet, a porcine model for HDRs has not been developed to date, which would gain recognition for hemodialysis system screening and mechanistic studies on HDR.

The aim of the present study was to fill this gap in preclinical research by establishing a pig model to explore the HDRs. To reach this goal, we used two types of dialysis membranes; FX CorDiax 40 (FXC) from Fresenius, and SureFlux-15UX (NSF) from Nipro, and to provide control for the membrane effects, a “sham dialysis” consisting of an extracorporeal circulation without dialyzer (Dialysis exchange-set, DES). A 60 min effective HD protocol was followed by the rinsing back of the extracorporeal blood into the circulation at the end of the procedure, to mimic the human therapy. Finally, the alternative complement pathway activator zymosan was used as positive control, which also activates innate immune cells *via* Toll-like receptors [20,21].

## Materials and methods

### Materials

FX CorDiax 40 dialyzer cartridges containing polysulfone/polyvinylpyrrolidone filters (FXC) and Dialysis exchange-set (DES), i.e., bypass tubing without membrane, were from Fresenius Medical Care (Bad Homburg, Germany). SureFlux-15UX cartridges, containing cellulose-triacetate filters (NSF), was from Nipro Corporation (Osaka, Japan). Forane (isoflurane) was from Rompharm (Otopen, Romania), and zymosan from Sigma-Aldrich (Budapest, Hungary).

### Experimental model

**Figure 1.**
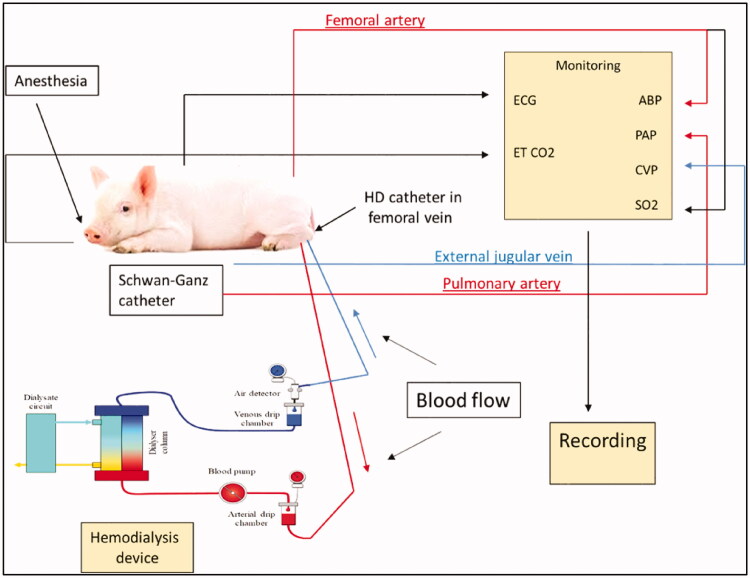
Experimental arrangement of porcine CARPA model combined with Hemodialysis (HD) device. The animal was anesthetized, and spontaneously ventilated. It was connected to the HD device as follows: the HD catheter originating from one femoral vein, blood flowing through the Dialyzer, and returning to the same femoral vein *via* a double lumen catheter. Systemic arterial blood pressure was measured *via* a femoral arterial catheter. Pulmonary arterial pressure was measured *via* a Swan-Ganz catheter through the jugular vein, advanced into the pulmonary artery. Abbreviations: ECG: electrocardiogram; ET CO2: end tidal partial pressure of carbon-dioxide; ABP: arterial blood pressure; PAP: pulmonary arterial pressure; CVP: central venous pressure; SO2: oxygen saturation.

Isovolemic (no-ultrafiltration) on-line post-dilution hemodiafiltration (HDF) was performed in anesthetized pigs (isoflurane 1.5%–2% in O_2_ flow) using 4008 S online plus dialysis equipment (Fresenius Medical Care). The experimental arrangement is shown on Figure 1. For dialysis a double lumen proVencare catheter (FDC-1125, Fr 11 diameter and 250 mm length) was introduced *via* the right femoral vein of pigs. The effects of a 60 min HDF treatment was followed by the reinfusion of blood from the extracorporeal circuit into the circulation over 10 min, and after a further 10 min waiting period by a bolus injection of 0.1 mg/kg zymosan, used as positive control for complement activation. Blood samples were then taken for further 30 min.

Upon HD, the blood and dialysate flow rates were 150 and 500 mL/min, respectively. The substitution fluid flow rate was set at 15 mL/min. Priming was made with 5,000 IU heparin in one liter of priming solution (NaCl 0.9%) followed with 1 liter without heparin. A bicarbonate dialysate was used (Fresenius; Na^+^: 138 mmol/L; K^+^: 4 mmol/L; Ca^2+^: 1.5 mmol/L; Mg^2+^: 0.5 mmol/L; glucose: 1 g/L; bicarbonate: 32 mmol/L). Hemodialysis sessions were performed in isovolemic conditions (no weight loss) and using sterile, apyrogenic dialysis fluid. For the control group (“sham hemodialysis” without a dialyzer), the blood was circulated in the HD tubing without a dialyzer (Dialysis exchange-set, DES) within the same conditions.

### Laboratory assays

Blood samples were obtained and stored in EDTA tubes before dialysis (0 min) and then every 15 min for 60 min dialysis, as well as 5 and 10 min after starting the reinfusion at 60 min. Samples were analyzed for blood cell counts by Abacus hematology analyzer (Diatron MI PLC, Budapest, Hungary), and for TXB2 and sC5b-9 levels in plasma. The latter ones were prepared by immediate centrifugation of EDTA anticoagulated blood (TXB2 tubes also containing indomenthacin) at 2500×*g* for 15 min at 4 °C, followed by withdrawal of the supernatant and storing it in 1 mL aliquots at −70 °C until further analysis. TXB2 was measured by an ELISA kit from Cayman Chemicals (Ann Arbor, MI, USA) using a FLUOstar Omega microplate reader (BMG Labtech). Porcine sC5b-9 determination was performed as previously described [22]. In brief, microtiter plates were coated with mouse anti-human sC5b-9 ascites (clone aE11) and incubated for 1 h with plasma containing 10 mM EDTA. The second Ab, biotinylated mouse anti-human C6 (Quidel A219), was stained with streptavidin − horseradish peroxidase (HRP) using ABTS and H_2_O_2_ substrate.

### Statistical analysis

Statistical analysis was performed by GraphPad Prism software (GraphPad Software, La Jolla, CA, USA). Differences among the three treatment groups were analyzed by one-way ANOVA, and the significance of the effects relative to baseline were computed using Sidak's multiple comparisons test. Comparison of baseline and peak effects was performed using Student’s paired t-test. To study the correlation between PAP and TXB2 values Pearson correlation coefficient was used. A p-value of <0.05 was considered to be statistically significant.

## Results

### Complement activation during hemodialysis, rinsing back of extracorporeal blood and zymosan bolus challenge

**Figure 2.**
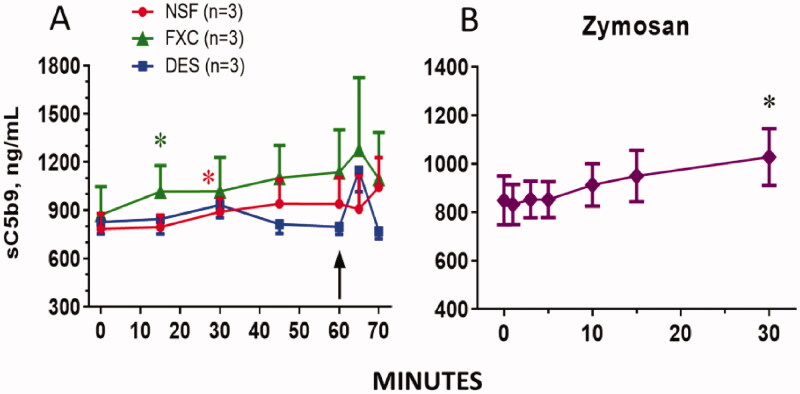
Plasma levels of the complement activation marker, sC5b-9, in pigs during HD for 60 min followed by blood reinfusion between 60 and 70 min (A), and following zymosan bolus, administered at 80 min. Bars are mean ± SD of *n* = 3 pigs in each group in panel A, and of 9 pigs in panel B. *, significant difference according to ANOVA (*p* < 0.05) relative to baseline. Key: NSF, FXC and DES mean Nipro SureFlux-15UX, Fresenius FX CorDiax 40 membranes and Dialysis exchange-set without filter membrane, respectively.

Hemodialysis using both NSF and FXC membranes resulted in a steady increase in complement activation as measured by sC5b-9 during the length of the dialysis (1 h), the rise reaching significance relative to baseline at 15 and 30 min in cases of FXC and NSF membranes, respectively (Figure 2(A)). Although the elevation of sC5b-9 in NSF membranes tends to be lower, no significant difference between groups has been found. In contrast, blood flow through the DES did not cause C activation (Figure 2(A)). Reinfusion of blood triggered a transient minor spike in sC5b-9 in case of FXC and DES animals. The complement-activating positive control zymosan, given as i.v. bolus, showed a modest, but significant increase in sC5b-9 over 30 min in all animals, enabling the pooling of these data in Figure 2(B).

Overall, the above data suggest the presence of complement activation during HD, entailing a modest level of terminal complex (sC5b-9) formation. To assess the biological consequences of this activation, we measured three known physiological effects of complement activation; blood cell changes, cardiopulmonary distress and thromboxane A2 release into the blood.

### Blood cell changes during hemodialysis, rinsing back of extracorporeal blood and zymosan challenge

**Figure 3.**
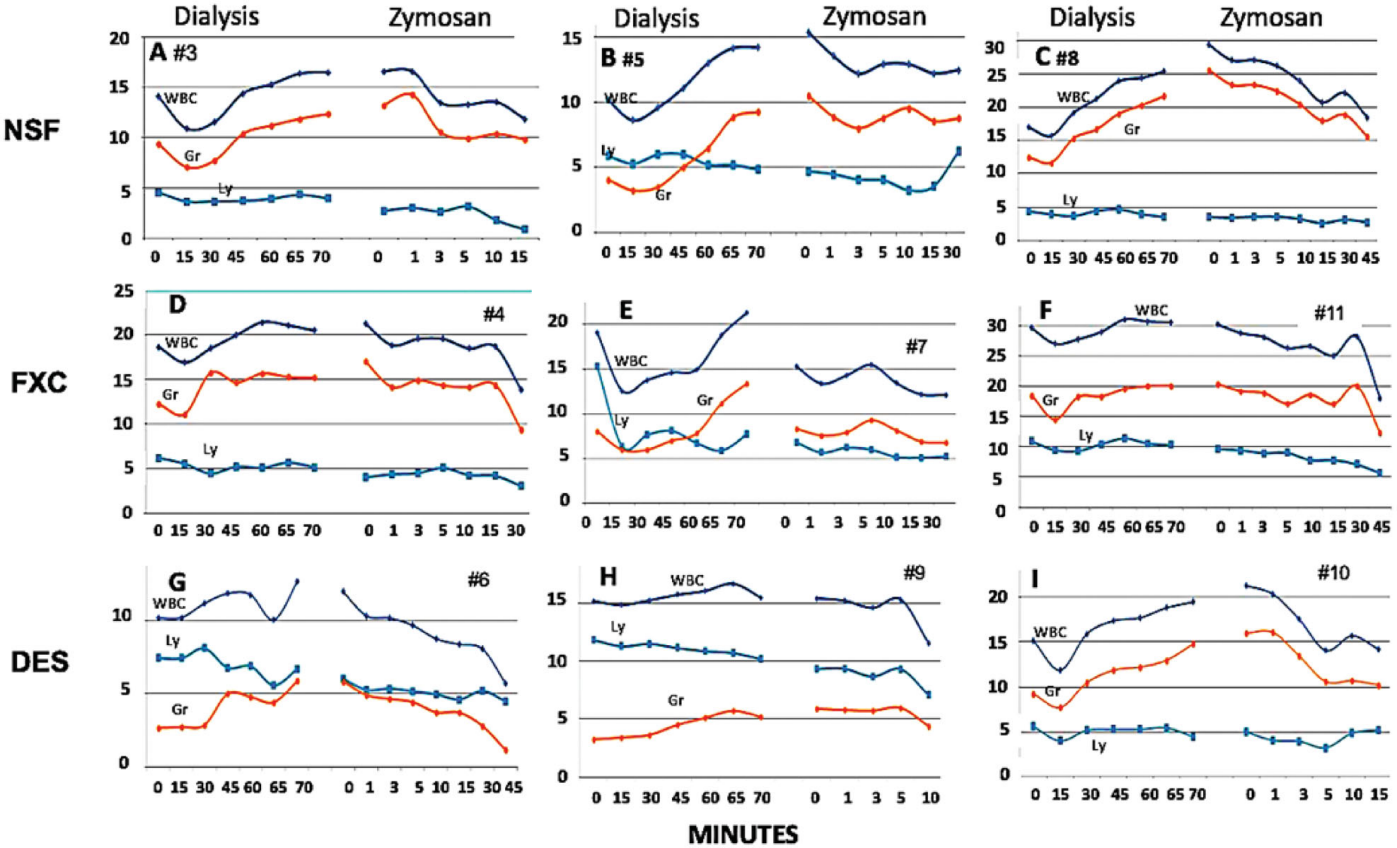
White blood cell (WBC) changes during HD (up to 60 min), reinfusion of extracorporeal blood (for 10 min starting at 60 min) and after bolus injection of zymosan started 10 min after blood reinfusion. The time versus blood cell count plots in the different panels show absolute counts (x 1,000) in individual pigs (#No. denotes experimental animals), 3-3 animals used in the NSF (A-C), FXC (D-F), and DES (G-I) groups. Different colors represent different cells, as labeled. DES, FXC and NSF are treatment groups defined in Figure 2. Abbreviations: WBC: white blood cell; Ly: lymphocyte; Gr: granulocyte.

We observed 10-40% leukopenia within 15 min in all animals undergoing dialysis (Figure 3(A–F)), and in one of the three sham-dialyzed pigs (Figure 3(I)). The secondary leukocytosis that followed the drop of leukocytes in these pigs is a typical sign of complement activation. Reinfusion of the extracorporeal blood into the circulation in the 60–70 min period did not trigger any abrupt change in these trends, although a membrane-independent slight acceleration of leukocytosis was observed in some pigs (Figure 3(A–C,E)). Based on the paralleling changes of WBC and granulocyte counts, these alterations represented mainly granulocyte response, while lymphocytes displayed only minor changes without a consistent trend.

In keeping with the lack of blood cell changes in 2 of 3 pigs in the DES group, averaging the maximal WBC changes indicated more expressed changes during HD of FXC and NSF animals (15%–17%) than in sham HD (DES, 4%) (Table 1), although the changes were statistically not significant. The platelet counts also suggested a trend for thrombocytopenia during HD, which was most expressed in the NSF group, particularly after zymosan treatment (bold italicized entries in Table 1). However, like with WBC, averaging the maximal drops of platelet counts showed no significant differences among the groups (Table 1). The red blood cell (RBC) counts, and plasma hemoglobin levels did not show any changes in any group (data not shown).

**Table 1.** Maximal WBC and platelet changes in pigs during dialysis, rinsing back of extracorporeal blood and zymosan treatment.

Phase	DES		FXC		NSF	
	Mean	SD (*n* = 3)	Mean	SD (*n* = 3)	Mean	SD (*n* = 3)
**WBC**						
Dialysis	95.5	15.9	82.5	14.4	84.9	7.7
Reinfusion	118.1	14.2	108.1	4.3	135.5	16.8
Zymosan	63.1	13.9	78.6	28.4	71.9	9.1
**Platelets**						
Dialysis	90.3	12.7	93.7	12.4	***81.3***	5.2
Reinfusion	107.5	9.3	101.5	6.7	95.2	6.9
Zymosan	92.7	15.5	93.5	8.3	***73.8***	9.9

DES, FXC and NSF are treatment groups defined in Figure 2. ANOVA showed no significant differences (*p* > 0.05) among the three groups in either blood cell count. However, the bold italicized entries in the NSF group suggest increase in thrombocytopenia (*p* > 0.05). Entries are % of baseline.

These complex WBC and platelet changes are consistent with dialysis membrane-induced complement activation during HD and, as expected, much stronger activation by zymosan. The presence of complement activation in the absence of HD membranes is ambiguous at this time, since the WBC changes in 1 out of 3 animals (Figure 3(I)) did suggest complement activation.

### Hemodynamic and blood pressure changes during hemodialysis, rinsing back of extracorporeal blood and zymosan bolus challenge

**Figure 4.**
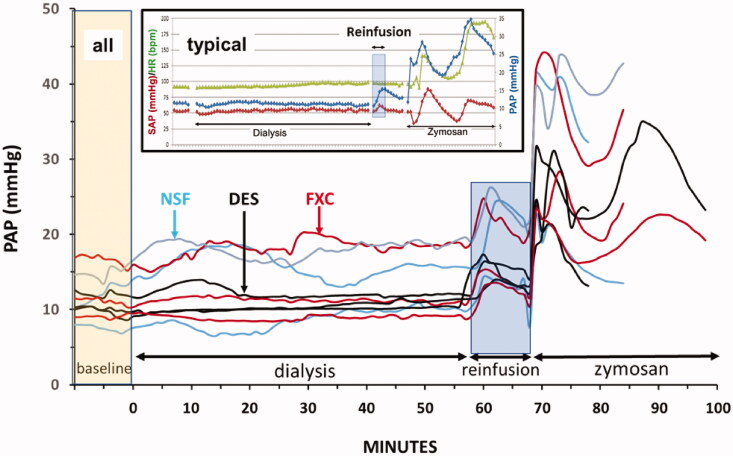
Individual recordings of the time course of PAP changes in pigs undergoing HD, extracorporeal blood reinfusion and bolus treatment with zymosan, as described in the Methods. The groups are color-coded: NSF (blue), FXC (red) and DES (black) pigs (*n* = 3, each). The insert illustrates the PAP (blue), SAP (red) and HR (black) changes in a typical (FXC) animal. The low number of animals and nonparametric variables made these differences statistically insignificant, but AUC calculations during HD confirmed these differences (not shown).

Figure 4 shows real-time tracing of PAP in all animals during all three phases of pig experiments: dialysis, reinfusion of extracorporeal blood, and zymosan bolus. We observed 5–10 mmHg rises of PAP starting within 15 min after the start of HD, peaking between 15 and 40 min, and returning to about 5 mm over baseline wherever such responses observed. The color-coding of individual pigs allows visual comparison of responses in the three groups, suggesting increased frequency and extent in the following order: DES < FXC < NS, i.e., the same order that was observed for C activation and blood cell changes in Figures 2 and 3.

Figure 4 also shows that blood reinfusion into pigs led to a 5–10 mmHg sudden rise of PAP in all animals which, unlike the pulmonary hypertension during HD, started at the same time (a few minutes after the start of reinfusion) and were remarkably similar in all animals in their rise and decline over 10 min (blue rectangle). These changes represented about 20% and 40% rises relative to the pre-dialysis and pre-reinfusion PAP values, respectively, and were highly significant relative to baseline (*p* < 0.001) (Table 2).

**Table 2.** Pulmonary hypertension following HD and blood rinsing back, expressed as percentage of pre-dialysis and pre-reinfusion baselines.

% increase in PAP							All groups		
Relative to	DES		FXC		NSF				
	Mean	SD (*n* = 3)	Mean	SD (*n* = 3)	Mean	SD (*n* = 3)	Mean	SD (*n* = 9)	*p*
Pre-dialysis	8.7	4.3	12.0	8.0	37.7	13.2	19.5	15.9	<0.001
Pre-reinfusion	29.7	7.3	45.8	11.0	45.4	11.8	40.3	11.9	
Pre-zymosan	–	–	–	–	–	–	146.5	38.8	<0.001

The % values mean the maximal PAP rise relative to the respective baseline values, computed with the formula: [PAP(max)/PAP(baseline)x100]-100, where the baselines are the pre-dialysis, pre-reinfusion, and pre-zymosan values. The mean%±SD values in the different groups were subjected to ANOVA in both comparisons, which was not significant (*p* > 0.05). The means ± SD values of pooled PAP showed highly significant rises (*p* < 0.001) relative to the baselines values (Student’s paired *t*-test).

Administration of zymosan 10 min after the end of blood reinfusion led to superimposition of massive mono-, or biphasic rises in PAP on reinfusion-induced pulmonary hypertension in all animals, lasting over 30 min. This effect indicated equal sensitivity of all pigs to immune-triggered pulmonary response, at the same time showing the biological limits of this response between 40-50 mmHg. Zymosan rose by about 150% relative to pre-zymosan PAP values that was highly significant (*p* < 0.001) (Table 2). Among these PAP changes the blood reinfusion-, and zymosan-induced ones were associated with paralleling, but less expressed changes of SAP and heart rate could be found (see insert in Figure 4).

### Plasma TXB2 changes during HD, rinsing back of extracorporeal blood and zymosan bolus challenge

Yet another biological consequence of complement activation in pigs is the release of TXA2 in blood, which was found to be a key mediator of liposomal C-activation-related pulmonary hypertension in pigs [23–26]. Thus, to explore the presence of an established mediator between complement activation and pulmonary hypertension in the case of HD, we performed serial TXB2 assays in pig blood during the 3 phases of experiments.

**Figure 5.**
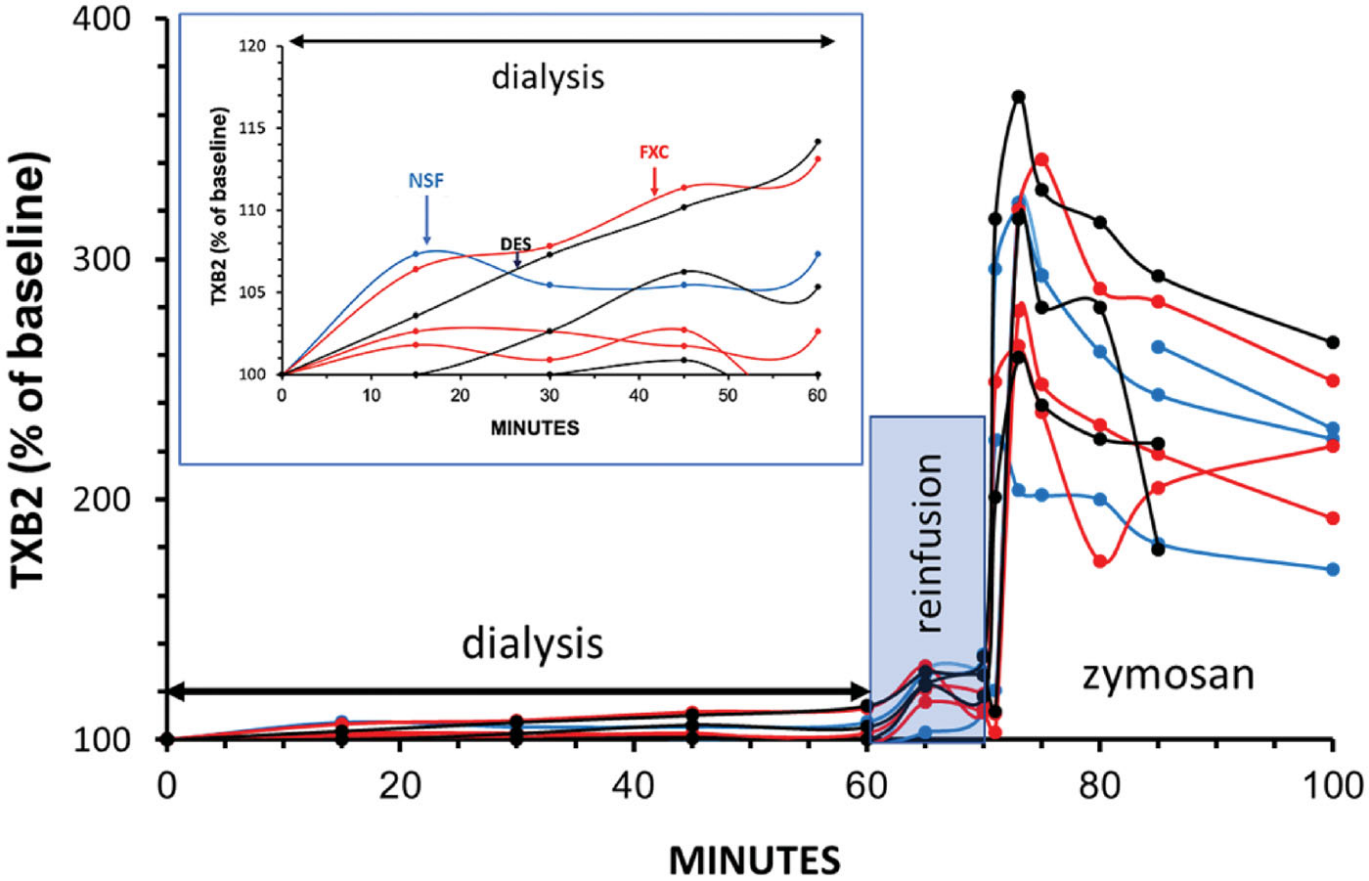
TXB2 changes in pigs undergoing dialysis, rinsing back of extracorporeal blood and bolus treatment with zymosan, expressed as % of baseline. The groups are color-coded: NSF (blue), FXC (red) and DES (black) pigs (*n* = 3, each). The insert amplifies the changes during HD.

The changes of TXB2 mimicked those of PAP inasmuch as during HD, only minor (<20%) rises of TXB2 were observed in those animals where C activation, blood cell changes and pulmonary hypertension were also observed (Figure 5 and insert in Figure 5). Thereafter, reinfusion of blood at 60 min led to sudden, 20%–40% increases of TXB2, giving rise of overlapping peaks preceding similar extent rises of PAP. Also, in perfect agreement with the changes in PAP, zymosan caused massive increases in TXB2 in all animals in the final 30 min. Accordingly, these TXB2 responses were highly significant relative to baseline (*p* < 0.0001) and were not different between the three groups (*p* > 0.05). Moreover, there was significant linear correlation between the % PAP and TXB2 levels (R^2^=0.82, *p* < 0.001) during the study (data not shown).

## Discussion

### Goals and rationale

The purpose of the present study was to establish a porcine model of HD reactions, which enables the comparison of various dialyzers (e.g. FXC and NSF) and provides a platform for exploring mechanisms of these reactions and identifying new approaches for their prevention. The rationale lies in the facts that 1) acute adverse hemodialysis reactions still represent an unpredictable and unsolved clinical issue, 2) pigs are sensitive for complement mediated HSRs that reproduce some of the main symptoms of dialysis reactions, and 3) hemodialysis membranes, and extracorporeal circuits in particular, can activate complement.

### Adverse hemodialysis reactions: influencing factors and mechanism

Hypersensitivity reactions represent a hazard to the clinical implementation of hemodialysis in some patients, yet the mechanism of the phenomenon is poorly understood. Part of the studies points to typical IgE-mediated allergy, one culprit being ethylene oxide [27–29] and another one is polyvinyl-pyrrolidone (PVP) [3]. Other evidence, in turn, suggest a non-IgE-mediated mechanism, namely complement activation. A study using cellulose (Cuprophan®) membranes, Suzuki et al. [30] showed a causal relationship between C3a and C5a anaphylatoxin levels in the arterial lines and the rise of anaphylactoid reactions in patients. Complement activation was shown to proceed *via* the alternative pathway and was linked with mast cell release reaction directly underlying the symptoms [31].

Reuse of dialyzers on patients having a history of an anaphylactoid reaction during HD suppressed not only the rise of C3a levels but also the recurrence of the reaction so-called ‘first-use syndrome’, suggesting that complement activation in used dialyzers was less intense than in new ones [32,33]. This observation raises the concept of ‘membrane passivation’ generated by preexposure and primed dialyzers to patients’ proteins (i.e., albumin and other proteins [34–36].

Interestingly, despite the development of advanced high-performance FX-CorDiax filters (Fresenius Medical Care) fitted with nanomodified polysulfone (PSu) dialysis membranes and steam sterilized dialyzers [37], hazard of HDRs could not be eliminated [3,38,39]. For example, Boer et al. documented dyspnea, hypotension, hypoxia, bronchospasm, chest pain, pruritus, urticaria and abdominal symptoms occurring in the 22%–69% range within 30 min after starting the HD in 30 patients dialyzed with PSu/polyethersulfone (PSu/PESu) membranes [39]. Beyond these mechanisms, contact activation on the membrane resulting in bradykinin generation, and dialysate contamination [40–42] are being discussed in the context of HDRs. However, it should be kept in mind that in addition to the dialyzer (storage condition) [43], dialysis parameters, such as flow rate and operating mode including rinse back procedure [44] as well as individual sensitivity to allergy are key determinants of HDRs [43,44]. These facts suggest that some hydrophilic modifiers of the PSu/Polyethersulfone membranes (i.e., PVP) may be released at some points in time and may trigger such HSRs in pre-sensitized patients [45].

Regardless of the trigger mechanism, HDRs can be a potentially life-threatening condition that leads to stop the dialysis session immediately without returning the blood to the patient [46]. In fact, not reinfusing the blood to the patient is an empirical and safety rule to avoid exacerbation of HDR [10], although the exact mechanism of this phenomenon had not been understood. As discussed later, one novelty of our study was to reproduce and substantiate this phenomenon in pigs, taking TXB2 and/or PAP as surrogate biomarkers of HDRs.

### Use of the porcine CARPA model to study dialysis reactions

In addition to the HSRs observed during cardiopulmonary bypass surgery [12–14] and apheresis [15–17], the symptoms of HDRs listed in the introduction are also characteristic of nanoparticle-induced infusion reactions, wherein complement activation has also been implicated as a major contributor mechanism [18,19,47]. These reactions, referred to as CARPA [18,19,47], are uniquely modeled in pigs, because the pulmonary circulation of pigs is abundant in intravascular macrophages (PIM cells), making this species extremely sensitive to the cardiopulmonary reactions associated with complement activation [48,49]. However, to date, only nanomedicine-induced HSRs have been studied in pigs, whereas other medical situations of iatrogenic HSRs, such as HDR, in this model has not yet been investigated.

The use of our pig model in studying ADRs is rationalized by the similarity of clinical picture, as some of the symptoms of human HDRs are also observed in porcine CARPA induced by other means [49–54]. Of key importance in this regard is that pigs can develop severe cardiopulmonary distress during CARPA, which is responsible for most fatal cases of HDR in man [50]. Thus, pigs enable us to study the clinically most serious, potentially lethal adverse effect of human HDR. Actually, the hemodynamic changes with a rise of PAP, which are key pathogenic factors in human cardiopulmonary distress during HSRs [50], are the most reproducible endpoints of porcine CARPA whose quantitation in numerous studies in the past [22–26,47–54] provide a rich database for comparison with HSRs caused by other treatments.

Nephrectomy is not part of our porcine CARPA model. Therefore, we need to exercise caution when drawing conclusions to uremic subjects (pigs or humans). It is known that end stadium kidney disease patients on chronic HD having chronic inflammation because of the activation of the immune system. Until now, we have not seen any clinical data that chronic uremia itself causes the activation of the complement system. Nevertheless, the patients on HD having complement system activation because of the hemodialysis materials, e.g. the dialyzer. Thereafter the complement system plays a crucial role in promoting inflammation, coagulation, and oxidative burst during an HD session. However, we believe that there would be no differences between uremic and non-uremic pigs when we investigated the complement activation by the dialyzer.

For all these reasons the use of pigs in the present study of ADRs may represent an important advance and useful tool to further explore these reactions.

### The implications of changes observed in the present pig study

Our pig study did not reveal intense symptoms of HSR during HDR, but rather a significant steady rise of sC5b-9, along with transient granulopenia followed by granulocytosis, and rise of TXB2 in plasma. These changes are consistent with constant, low-grade complement activation in the FXC and NSF groups. The dialysis-induced thrombocytopenia was more expressed with NSF membranes than FXC (Table 1), but these data are not adequately powered to assess differences between these membranes’ biocompatibility and safety. The severe reaction to zymosan at the end of the experiments in all pigs provided proof of equal immune competence of animals in the three different groups. These zymosan reactions could be observed, and used in all previous pig studies as positive control for CARPA [22–26,51–54].

### The effect of blood rinsing back

The unique, real surprise in this study came at the end of HD, after reinfusing the extracorporeal blood back into the animal’s circulation, whereupon we noted an abrupt rise of PAP in notable synchrony in all animals. To exclude a volume effect and confirm the immune origin of this observation, we also measured plasma TXB2, which showed remarkable parallelism with these PAP changes. In some, but not all animals, we also observed a transient spike of sC5b-9 (Figure 2(A)), and acceleration of compensatory leukocytosis (Figure 3), which suggests complement activation. Thus, abrupt changes can be explained by an anaphylatoxin spike superimposed on the membrane-induced activation, when the extracorporeal blood reaches the circulation, although significant correlation between the PAP/TXB2 spikes and sC5b-9 changes could not be established. This, however, does not definitely rule out a second wave of minor complement activation coinciding with blood reinfusion.

It should be noted regarding the sensitivity of porcine sC5b-9 ELISA that it is based on a cross-reactive human mAb (aE11), capturing a neoepitope exposed in activated C9 when C9 is incorporated in sC5b-9. This epitope is exposed in pigs but to a lesser extent than in humans [55], which is also shown by the relatively modest slope of sC5b-9 following zymosan injection. Since the increase in sC5b-9 caused by the dialysis membranes was comparable to the effect of zymosan, it is likely that complement activation was relatively more pronounced than evident from the absolute values of sC5b-9. Nevertheless, it did not trigger major pulmonary hypertension until a second activation trigger reached the immune system, implying that the anaphylatoxin release associated with membrane-induced complement activation remained subthreshold for pulmonary vasoreactivity.

Further information on complement proteins deposition could be gained from the analysis of the membrane eluate that has not been performed here. However, literature data show the clinical implications [56], as well as the *in vitro* secondary effects on dialyzer membranes [57] of complement activation. The role of sC5b-9 in human HDR is known for long; however, until its recent “rediscovery” it has been forgotten [56].

### The double hit theory of the mechanism

The observation on sudden pulmonary hypertension caused by blood reinfusion into the circulation can most easily rationalized by the “double hit” hypothesis, proposed for nanomedicine-induced infusion reactions [22,48,49,51,52]. This theory postulates complement activation and a subsequent binding of anaphylatoxins to their specific receptors on allergy-mediating innate immune cells (PIM cells in pigs; mast cells, basophils, macrophages and leukocytes in humans), coinciding with the binding of immune reactive nanoparticles to (an) other surface receptor(s) on these cells, known as pathogen-associated pattern recognition receptors, such as Toll-like receptors. The concurrent stimulation of these cells *via* these different surface receptors result in synergistic intracellular signaling for the release of inflammatory mediators, including TXA2.

In the case of the porcine model used in the present study the low degree complement activation may provide one “hit”, although the purported slow and extended formation of anaphylatoxins suggest rather a “priming” than a “hit”. As for the second “hit” on allergy mediating cells, at this time we can only speculate on their identity. Ansorge et al. have shown the accumulation of inflammatory mediators in blood during a prolonged contact with the dialyzer [58], but other causes cannot be excluded either. Possible culprits include hydrophilic membrane modifiers (i.e., PVP), polymer particles leaking from the tubing, undisrupted cellular aggregates, and free radicals formed during the stasis of blood. Anaphylatoxins could also build up during a stop-flow, because of extended interaction of complement proteins with the wall of the tubes and/or in the absence of metabolic and cellular removal in the body.

## Conclusions

In summary, our study highlights the utility of the pig CARPA model to study HDRs, wherein the most reproducible biomarker of adverse immune reactivity, pulmonary hypertension, is also a relevant clinical biomarker for HSR severity in man [59]. The “double hit” mechanism of HDR, revealed by the analysis of PAP, points to a critical role in HDR of blood flow and its possible pausing in the extracorporeal circuit, contraindicating flow-stop and the rinsing of blood back into the circulation following HD. Furthermore, we are not aware of previous data raising the possibility that low-degree complement activation may be an intrinsic feature of extracorporeal circuits regardless of the presence of dialyzer membranes, and that extracorporeal stagnation of blood may entail accumulation of immune reactive byproducts that contribute to HDR. Further studies in the model, also focusing on changes not measured here (oxygenation, bradykinin, platelets, etc.; see references [57–62]) will hopefully advance our understanding of HDRs.

## Declarations

The study was designed in accordance with accepted pharmacological principles in order to meet the requirements of the principles of Hungarian Act 1998: XXVIII regulating animal protection (latest modified by Act 2011 CLVIII) and in Government Decree 40/2013 on animal experiments. All procedures carried out on animals had been approved by the local ethical committee of the Semmelweis University.

## References

[1] Salem M, Ivanovich PT, Ing TS, et al. Adverse effects of dialyzers manifesting during the dialysis session. Nephrol Dial Transplant. 1994;9 (Suppl 2):127–137. 8065604

[2] Lodi CA, Vasta A, Hegbrant MA, et al. Multidisciplinary evaluation for severity of hazards applied to hemodialysis devices: an original risk analysis method. Clin J Am Soc Nephrol. 2010;5(11):2004–2017. 20813858 10.2215/CJN.01740210 PMC3001776

[3] Bacelar Marques ID, Pinheiro KF, de Freitas do Carmo LP, et al. Anaphylactic reaction induced by a polysulfone/polyvinylpyrrolidone membrane in the 10th session of hemodialysis with the same dialyzer. Hemodial Int. 2011;15(3):399–403. 21624039 10.1111/j.1542-4758.2011.00553.x

[4] Shu KH, Kao TW, Chiang WC, Wu VC. A case of anaphylactic shock induced by FX60 polysulfone hemodialyzer but not F6-HPS polysulfone hemodialyzer. Hemodial Int. 2014;18(4):841–845. 24923810 10.1111/hdi.12184

[5] Kliger AS. Maintaining safety in the dialysis facility. Clin J Am Soc Nephrol. 2015;10(4):688–695. 25376767 10.2215/CJN.08960914 PMC4386259

[6] Liyanage T, Ninomiya T, Jha V, et al. Worldwide access to treatment for end-stage kidney disease: a systematic review. Lancet. 2015;385(9981):1975–1982. 25777665 10.1016/S0140-6736(14)61601-9

[7] Sayeed K, Murdakes C, Spec A, et al. Anaphylactic shock at the beginning of hemodialysis. Semin Dial. 2016;29(1):81–84. 26538311 10.1111/sdi.12449

[8] Butani L, Calogiuri G. Hypersensitivity reactions in patients receiving hemodialysis. Ann Allergy Asthma Immunol. 2017;118(6):680–684. 28456484 10.1016/j.anai.2017.04.006

[9] Szebeni J, Fishbane S, Hedenus M, et al. Hypersensitivity to intravenous iron: classification, terminology, mechanisms and management. Br J Pharmacol. 2015;172(21):5025–5036. 26265306 10.1111/bph.13268 PMC4687801

[10] Saha M, Allon M. Diagnosis, treatment, and prevention of hemodialysis emergencies. Clin J Am Soc Nephrol. 2017;12(2):357–369. 27831511 10.2215/CJN.05260516 PMC5293333

[11] Schmaier AH. The contact activation and kallikrein/kinin systems: pathophysiologic and physiologic activities. J Thromb Haemost. 2016;14(1):28–39. 26565070 10.1111/jth.13194

[12] Utley JR. Pathophysiology of cardiopulmonary bypass: current issues. J Card Surg. 1990;5(3):177–189. 2133841 10.1111/j.1540-8191.1990.tb01036.x

[13] Weiler JM, Gellhaus MA, Carter JG, et al. A prospective study of the risk of an immediate adverse reaction to protamine sulfate during cardiopulmonary bypass surgery. J Allergy Clin Immunol. 1990;85(4):713–719. 2182695 10.1016/0091-6749(90)90189-b

[14] Tamim M, Demircin M, Guvener M, et al. Heparin-coated circuits reduce complement activation and inflammatory response to cardiopulmonary bypass. Panminerva Med. 1999;41(3):193–198. 10568115

[15] Rubenstein MD, Wall RT, Wood GS, et al. Complications of therapeutic apheresis, including a fatal case with pulmonary vascular occlusion. Am J Med. 1983;75(1):171–174. 6859081 10.1016/0002-9343(83)91184-1

[16] Rosenkvist J, Berkowicz A, Holsoe E, et al. Plasma exchange in myasthenia gravis complicated with complement activation and urticarial reactions using fresh-frozen plasma as replacement solution. Vox Sang. 1984;46(1):13–18. 6702139 10.1111/j.1423-0410.1984.tb00042.x

[17] Savage WJ, Savage JH, Tobian AA, et al. Allergic agonists in apheresis platelet products are associated with allergic transfusion reactions. Transfusion. 2012;52(3):575–581. 21883267 10.1111/j.1537-2995.2011.03310.x PMC3711211

[18] Szebeni J. Complement activation-related pseudoallergy: a stress reaction in blood triggered by nanomedicines and biologicals. Mol Immunol. 2014;61(2):163–173. 25124145 10.1016/j.molimm.2014.06.038

[19] Szebeni J, Simberg D, Gonzalez-Fernandez A, et al. Roadmap and strategy for overcoming infusion reactions to nanomedicines. Nat Nanotechnol. 2018;13(12):1100–1108. 30348955 10.1038/s41565-018-0273-1 PMC6320688

[20] Sato M, Sano H, Iwaki D, et al. Direct binding of toll-like receptor 2 to zymosan, and zymosan-induced NF-kappa B activation and TNF-alpha secretion are down-regulated by lung collectin surfactant protein A. J Immunol. 2003;171(1):417–425. 12817025 10.4049/jimmunol.171.1.417

[21] Volman TJ, Hendriks T, Goris RJ. Zymosan-induced generalized inflammation: experimental studies into mechanisms leading to multiple organ dysfunction syndrome. Shock. 2005;23(4):291–297. 15803050 10.1097/01.shk.0000155350.95435.28

[22] Kozma GT, Meszaros T, Vashegyi I, et al. Pseudo-anaphylaxis to polyethylene glycol (PEG)-coated liposomes: roles of anti-PEG IgM and complement activation in a porcine model of human infusion reactions. ACS Nano. 2019;13(8):9315–9324. 31348638 10.1021/acsnano.9b03942

[23] Szebeni J, Fontana JL, Wassef NM, et al. Hemodynamic changes induced by liposomes and liposome-encapsulated hemoglobin in pigs: a model for pseudoallergic cardiopulmonary reactions to liposomes. Role of complement and inhibition by soluble CR1 and anti-C5a antibody. Circulation. 1999;99(17):2302–2309. 10226097 10.1161/01.cir.99.17.2302

[24] Jackman JA, Meszaros T, Fulop T, et al. Comparison of complement activation-related pseudoallergy in miniature and domestic pigs: foundation of a validatable immune toxicity model. Nanomedicine. 2016;12(4):933–943. 26767512 10.1016/j.nano.2015.12.377

[25] Meszaros T, Kozma GT, Shimizu T, et al. Involvement of complement activation in the pulmonary vasoactivity of polystyrene nanoparticles in pigs: unique surface properties underlying alternative pathway activation and instant opsonization. Int J Nanomedicine. 2018;13:6345–6357. 30349254 10.2147/IJN.S161369 PMC6187999

[26] Fulop T, Kozma GT, Vashegyi I, et al. Liposome-induced hypersensitivity reactions: Risk reduction by design of safe infusion protocols in pigs. J Control Release. 2019;309:333–338. 31295544 10.1016/j.jconrel.2019.07.005

[27] Foley RJ, Reeves WB. Acute anaphylactoid reactions in hemodialysis. Am J Kidney Dis. 1985;5(2):132–135. 3970019 10.1016/s0272-6386(85)80010-x

[28] Lemke HD. Mediation of hypersensitivity reactions during hemodialysis by IgE antibodies against ethylene oxide. Artif Organs. 1987;11(2):104–110. 3297007 10.1111/j.1525-1594.1987.tb02639.x

[29] Villarroel F, Ciarkowski AAA. A survey on hypersensitivity reactions in hemodialysis. Artif Organs. 1985;9(3):231–238. 4051817 10.1111/j.1525-1594.1985.tb04384.x

[30] Suzuki Y, Uchida J, Tsuji H, et al. Acute changes in C3a and C5a in an anaphylactoid reaction in hemodialysis patients. Tohoku J Exp Med. 1987;152(1):35–45. 3497472 10.1620/tjem.152.35

[31] Rodriguez-Sanz A, Sanchez-Villanueva R, Dominguez-Ortega J, et al. Mechanisms involved in hypersensitivity reactions to polysulfone hemodialysis membranes. Artif Organs. 2017; 41(11):E285–E295. [10.1111/aor.12954] 28722144

[32] Daugirdas JT, Ing TS. First-use reactions during hemodialysis: a definition of subtypes. Kidney Int Suppl. 1988; 24:S37–S43. 2966256

[33] Pollak VE, Charoenpanich R, Robson M, et al. Dialyzer membranes: syndromes associated with first use and effects of multiple use. Kidney Int Suppl. 1988; 24:S49–S52. 2966257

[34] Clark WR, Macias WL, Molitoris BA, et al. Plasma protein adsorption to highly permeable hemodialysis membranes. Kidney Int. 1995; 48(2):481–488. 7564116 10.1038/ki.1995.317

[35] Vanommeslaeghe F, De Somer F, Josipovic I, et al. Evaluation of different dialyzers and the Impact of predialysis albumin priming in intermittent hemodialysis with reduced anticoagulation. Kidney Int Rep. 2019; 4(11):1538–1545. 31890995 10.1016/j.ekir.2019.07.010 PMC6933477

[36] Nakamura Y, Ozawa K, Akiba T, et al. Modified leukopenic response and complement activation during dialyzer reuse. Proc Clin Dial Transplant Forum. 1980;10:237–239. PMID: 6810348. 6810348

[37] Ronco C, Bowry SK, Brendolan A, et al. Hemodialyzer: from macro-design to membrane nanostructure; the case of the FX-class of hemodialyzers. Kidney Int Suppl. 2002;80:126–142. 10.1046/j.1523-1755.61.s80.23.x 11982827

[38] Arenas MD, Gil MT, Carreton MA, et al. Adverse reactions to polysulphone membrane dialyzers during hemodialysis. Nefrologia. 2007;27(5):638–642. 18045043

[39] Boer WH, Liem Y, de Beus E, et al. Acute reactions to polysulfone/polyethersulfone dialysers: literature review and management. Neth J Med. 2017;75(1):4–13. 28124665

[40] Yang RC, Lindsay RM. Dialyzer reactions in a patient switching from peritoneal dialysis to hemodialysis. Hemodial Int. 2005;9(2):120–126. 16191059 10.1111/j.1492-7535.2005.01123.x

[41] Montagnac R, Schillinger F, Milcent T, et al. Hypersensitivity reactions during hemodialysis. Role of high permeability, retrofiltration and bacterial contamination of the dialysate. Nephrologie. 1988;9(1):29–32. 3134622

[42] Parnes EL, Shapiro WB. Anaphylactoid reactions in hemodialysis patients treated with the AN69 dialyzer. Kidney Int. 1991;40(6):1148–1152. 1762316 10.1038/ki.1991.327

[43] Namekawa K, Kaneko A, Sakai K, et al. Longer storage of dialyzers increases elution of poly(N-vinyl-2-pyrrolidone) from polysulfone-group dialysis membranes. J Artif Organs. 2011; 14(1):52–57. 21286768 10.1007/s10047-011-0552-1

[44] Matsuda M, Sato M, Sakata H, et al. Effects of fluid flow on elution of hydrophilic modifier from dialysis membrane surfaces. J Artif Organs. 2008;11(3):148–155. 18836876 10.1007/s10047-008-0417-4

[45] Konishi S, Fukunaga A, Yamashita H, et al. Eluted substances from hemodialysis membranes elicit positive skin prick tests in bioincompatible patients. Artif Organs. 2015; 39(4):343–351. 25327279 10.1111/aor.12392

[46] Ebo DG, Bosmans JL, Couttenye MM, et al. Haemodialysis-associated anaphylactic and anaphylactoid reactions. Allergy. 2006;61(2):211–220. 16409199 10.1111/j.1398-9995.2006.00982.x

[47] Szebeni J. Complement activation-related pseudoallergy: a new class of drug-induced acute immune toxicity. Toxicology. 2005;216(2-3):106–121. 16140450 10.1016/j.tox.2005.07.023

[48] Szebeni J. Mechanism of nanoparticle-induced hypersensitivity in pigs: complement or not complement? Drug Discov Today. 2018;23(3):487–492. 29326077 10.1016/j.drudis.2018.01.025

[49] Szebeni J, Bawa R. Human clinical relevance of the porcine model of pseudoallergic infusion reactions. Biomedicines. 2020;8(4):82. 10.3390/biomedicines8040082 PMC7235862 32276476

[50] Kiykim AA, Horoz M, Ozcan T, et al. Pulmonary hypertension in hemodialysis patients without arteriovenous fistula: the effect of dialyzer composition. Ren Fail. 2010;32(10):1148–1152. 20954973 10.3109/0886022X.2010.516854

[51] Szebeni J, Bedőcs P, Csukás D, et al. A porcine model of complement-mediated infusion reactions to drug carrier nanosystems and other medicines. Adv Drug Deliv Rev. 2012;64(15):1706–1716. 22820530 10.1016/j.addr.2012.07.005

[52] Urbanics R, Bedőcs P, Szebeni J. Lessons learned from the porcine CARPA model: constant and variable responses to different nanomedicines and administration protocols. Eur J Nanomedicine. 2015;7:219–231.

[53] Szebeni J, Baranyi L, Savay S, et al. Complement activation-related cardiac anaphylaxis in pigs: role of C5a anaphylatoxin and adenosine in liposome-induced abnormalities in ECG and heart function. Am J Physiol Heart Circ Physiol. 2006;290(3):H1050–8. 16214844 10.1152/ajpheart.00622.2005

[54] Szebeni J, Bedocs P, Rozsnyay Z, et al. Liposome-induced complement activation and related cardiopulmonary distress in pigs: factors promoting reactogenicity of doxil and AmBisome. Nanomedicine. 2012;8(2):176–184. 21704590 10.1016/j.nano.2011.06.003

[55] Mollnes TE, Redl H, Høgåsen K, et al. Complement activation in septic baboons detected by neoepitope-specific assays for C3b/iC3b/C3c, C5a and the terminal C5b-9 complement complex (TCC). Clin Exp Immunol. 1993; 91(2):295–300. 7679061 10.1111/j.1365-2249.1993.tb05898.x PMC1554676

[56] Poppelaars F, Faria B, Gaya da Costa M, et al. The complement system in dialysis: a forgotten story? Front Immunol. 2018; 9:71. 29422906 10.3389/fimmu.2018.00071 PMC5788899

[57] Melchior P, Erlenkötter A, Zawada AM, et al. Complement activation by dialysis membranes and its association with secondary membrane formation and surface charge. Artif Organs. 2021;45(7):770–778. 33326619 10.1111/aor.13887

[58] Ansorge W, Pelger M, Dietrich W, et al. Ethylene oxide in dialyzer rinsing fluid: effect of rinsing technique, dialyzer storage time, and potting compound. Artif Organs. 1987;11(2):118–122. 3593041 10.1111/j.1525-1594.1987.tb02641.x

[59] Kawar B, Ellam T, Jackson C, et al. Pulmonary hypertension in renal disease: epidemiology, potential mechanisms and implications. Am J Nephrol. 2013;37(3):281–290. 23548763 10.1159/000348804

[60] Campos I, Chan L, Zhang H, et al. Intradialytic hypoxemia in chronic hemodialysis patients. Blood Purif. 2016;41(1-3):177–187. 26765143 10.1159/000441271 PMC6109968

[61] Kooman JP, Stenvinkel P, Shiels PG, et al. The oxygen cascade in patients treated with hemodialysis and native high-altitude dwellers: lessons from extreme physiology to benefit patients with end-stage renal disease. Am J Physiol Renal Physiol. 2021;320(3):F249–F61. 33356957 10.1152/ajprenal.00540.2020

[62] De Sanctis LB, Stefoni S, Cianciolo G, et al. Effect of different dialysis membranes on platelet function. A tool for biocompatibility evaluation. Int J Artif Organs. 1996;19(7):404–410. 8841854

